# The prospective associations of 24-hour movement behaviors and domain-specific activities with executive function and academic achievement among school-aged children in Singapore

**DOI:** 10.3389/fpubh.2024.1412634

**Published:** 2024-09-04

**Authors:** Natarajan Padmapriya, Jonathan Y. Bernard, Sarah Yi Xuan Tan, Anne H. Y. Chu, Claire Marie Jie Lin Goh, Shuen Lin Tan, Lynette P. Shek, Yap Seng Chong, Kok Hian Tan, Shiao-Yng Chan, Fabian Yap, Keith M. Godfrey, Yung Seng Lee, Michael J. Meaney, Johan G. Eriksson, Chuen Seng Tan, Evelyn C. Law, Falk Müller-Riemenschneider

**Affiliations:** ^1^Saw Swee Hock School of Public Health, National University of Singapore, Singapore, Singapore; ^2^Department of Obstetrics & Gynaecology and Human Potential Translational Research Programme, Yong Loo Lin School of Medicine, National University of Singapore, Singapore, Singapore; ^3^Singapore Institute for Clinical Sciences (SICS), Agency for Science, Technology and Research (A*STAR), Singapore, Singapore; ^4^Université Paris Cité and Université Sorbonne Paris Nord, Inserm, INRAE, Centre for Research in Epidemiology and StatisticS (CRESS), Paris, France; ^5^Department of Paediatrics, Yong Loo Lin School of Medicine, National University of Singapore, Singapore, Singapore; ^6^Khoo Teck Puat-National University Children’s Medical Institute, National University Health System, Singapore, Singapore; ^7^KK Women’s and Children’s Hospital, Singapore, Singapore; ^8^Duke-National University of Singapore, Singapore, Singapore; ^9^Lee Kong Chian School of Medicine, Nanyang Technological University, Singapore, Singapore; ^10^Medical Research Council Lifecourse Epidemiology Centre, University of Southampton, Southampton, United Kingdom; ^11^NIHR Southampton Biomedical Research Centre, University of Southampton and University Hospital Southampton NHS Foundation Trust, Southampton, United Kingdom; ^12^Ludmer Centre for Neuroinformatics and Mental Health, Department of Psychiatry, Douglas Mental Health University Research Centre, McGill University, Montreal, QC, Canada; ^13^Department of General Practice and Primary Health Care, University of Helsinki and Folkhälsan Research Center, Helsinki, Finland; ^14^Digital Health Center, Berlin Institute of Health, Charité-Universitätsmedizin Berlin, Berlin, Germany

**Keywords:** physical activity, sedentary behavior, sleep, movement behaviors, cognition, children, accelerometer, compositional data analysis

## Abstract

**Background:**

Physical activity (PA), sedentary behavior (SB), and sleep are collectively referred to as 24-h movement behaviors, which may be linked to cognitive development in children. However, most of the evidence was based on cross-sectional studies and/or solely relied on parent-reported information on children’s behaviors, and it remains uncertain whether all domains/contexts of PA and SB are similarly associated with executive function and academic achievement.

**Objective:**

We investigated the prospective associations of accelerometer-measured 24 h-movement behaviors and domain-specific PA and SB with executive function and academic achievement among school-aged children in Singapore.

**Methods:**

The Growing Up in Singapore Toward healthy Outcomes (GUSTO) cohort used a wrist-worn accelerometer (Actigraph-GT3x+) to measure 24 h-movement behaviors data at ages 5.5 and 8 years. Executive function and academic achievement were assessed using NEuroPSYchology (NEPSY) and Wechsler Individual Achievement Tests at ages 8.5 and 9-years, respectively. Compositional data analyses were conducted to explore the associations of 24 h-movement behavior with outcomes, and multiple linear regression models to examine the associations of domain-specific PA and SB with outcomes (*n* = 432).

**Results:**

Among 432 children whose parents agreed to cognitive assessments (47% girls and 58% Chinese), the composition of 24 h-movement behaviors at ages 5.5 and 8 years was not associated with executive function and academic achievement. However, higher moderate-to-vigorous PA (MVPA) relative to remaining movement behaviors at age 5.5 years was associated with lower academic achievement [Mean difference (95% confidence interval): −0.367 (−0.726, −0.009) z-score], and reallocating MVPA time to sleep showed higher academic achievement scores [30 min from MVPA to sleep: 0.214 (0.023, 0.404) z-score]. Certain domains of PA and SB, notably organized PA/sports, outdoor play, and reading books were favorably associated with outcomes of interest, while indoor play and screen-viewing were unfavorably associated.

**Conclusion:**

The associations between movement behaviors and cognitive outcomes are multifaceted, influenced by specific domains of PA and SB. This study underscores the importance of participation in organized PA/sports, outdoor active play, and reading books, while ensuring adequate sleep and limiting screen viewing, to enhance cognitive outcomes. These findings underscore the need for further research into time-use trade-offs. Such studies could have major implications for revising current guidelines or strategies aimed at promoting healthier 24 h-movement behaviors in children.

**Study registration:**

https://clinicaltrials.gov/, NCT01174875.

## Introduction

Executive function comprises a set of higher-order cognitive skills, including working memory (i.e., retaining mentally manipulating information), inhibitory control (i.e., resisting impulsive behavior), and cognitive flexibility (i.e., switching attention). Executive function regulates complex behaviors, and is essential in goal-directed actions such as decision-making, school adjustment, and academic achievement (1–3). Deficits in executive function during childhood may be associated with psychopathological symptoms, including anxiety, depression and cognitive decline later in life (4). Academic functioning in children is measured by their success in achieving academic goals, such as the ability to solve arithmetic problems, spell, and read fluently (5). Academic achievement is closely linked to an individual’s self-esteem, well-being and socio-economic growth (5–7).

Increasing evidence asserts that a child’s brain is sensitive to physical activity (PA), sedentary behavior (SB), and sleep (3, 5, 8). However, studies examining the associations of PA with executive function and/or academic performance among children aged 5 to 18 years have reported inconclusive findings. Although many recent reviews suggested positive associations (9–13), a significant proportion of research remains inconclusive. An earlier systematic review revealed that nearly half of all investigations found no associations (*n* = 251), and some (1.5%) even showed inverse associations (14). Moreover, the associations of PA with cognitive outcomes may vary depending on the domains of PA, such as organized sports, active play, active commuting, and/or indoor activities (15–17). Emerging evidence on the associations of SB with executive function and learning among children aged 5 to 14 years remains inconclusive (18–21). Previous studies focused on television (TV) time as the main proxy for leisure-time SB (19, 21, 22), while research on handheld screen use (including mobile phones and tablets), non-screen SB, and total SB, such as reading books, playing while sitting and passive commuting, is scarce (19, 21, 23). The mixed evidence suggests that the associations of PA and SB with executive function and/or academic achievement may be complex and influenced by various factors (24). Therefore, further research is warranted to investigate the relationships of time spent in total and domain/context-specific PA and SB with cognitive development among children. Sleep is also a critical aspect of a child’s development and plays a crucial role in executive function (25). Previous studies were largely conducted in children with sleep disorders (26–28). Growing evidence suggests that sleep duration may also be positively associated with executive function and academic achievement in normally developing children aged 3 to 14-years (29–34).

Current evidence is largely derived from cross-sectional studies and depends on self-or proxy-reported data for movement behaviors, and longitudinal studies that used device-measured movement behaviors are limited (14, 18–21, 34). During a 24-h period, children are continuously engaging in either light-intensity PA (LPA), moderate-to-vigorous-intensity physical activity (MVPA), SB, or sleep. These behaviors are collectively known as 24-h movement behavior (35). Changes in the time spent in one behavior inevitably result in changes in one or more other behaviors (35). Advances in movement behavior research propose the utilization of compositional data analysis (CoDA) to consider the interdependence between the allocations of time in different movement behaviors (36). To date, only one cross-sectional study examined the full-spectrum of 24 h-movement behaviors on preschool-aged children’s executive function and adequately accounted for interdependency of 24 h-movement behaviors (37). Elucidating prospective associations of the full spectrum of 24 h-movement behavior and various domains of PA and SB with cognitive outcomes may be key to determining the most effective strategies for promoting these behaviors and optimizing cognitive development in children. Considering the aforementioned gaps in the current literature, this prospective cohort study examined the associations of accelerometer-measured 24 h-movement behaviors, and domain-specific PA and SB with executive function and academic achievement among school-aged children in Singapore.

## Methods

### Study design

**Figure 1:**
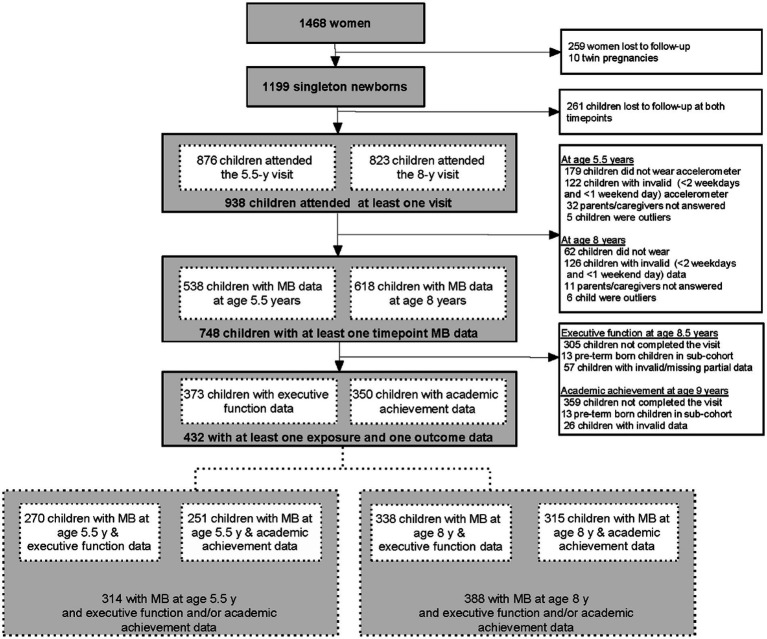
Flowchart of the participants of the study. MB, movement behavior.

The Growing Up in Singapore Toward healthy Outcomes (GUSTO) is an ongoing Asian mother–child cohort study that aims to comprehensively phenotype mother–child dyads from pregnancy until adolescence and beyond (38). The GUSTO study recruited pregnant women aged at least 18 years, of Chinese, Malay, or Indian ethnicity, who attended their first-trimester antenatal visit at one of two major public maternity units in Singapore, namely KK Women’s and Children’s Hospital (KKH) or National University Hospital (NUH), between June 2009 and September 2010. Singaporean citizens or permanent residents planning to deliver at KKH or NUH, with the intention of remaining in Singapore for at least the next 5 years, belonging to the major ethnic groups (Chinese, Malay, and Indian), and willing to donate birth tissues were included in the GUSTO study. Women who were receiving chemotherapy, psychotropic drugs, or had type I diabetes mellitus were excluded from the GUSTO study. Additionally, twin pregnancies were excluded from the present study. In total, 1,450 women were recruited at their first trimester of pregnancy and 18 women were recruited at delivery, and 1,199 singleton babies were born and enrolled. A subset of parents in the GUSTO study agreed to cognitive assessments for their children, and these children with valid data were included in the present study (*n* = 432) (Figure 1). The study was approved by the SingHealth Centralized Institutional Review Board and the National Healthcare Group Domain Specific Review Board (ClinicalTrials.gov: NCT01174875), and all participants provided written informed consent.

### Accelerometer-measured 24-h movement behaviors

We used the ActiGraph GT3X+ (ActiGraph Inc., Pensacola, FL), a triaxial accelerometer, to collect raw acceleration data. Children were instructed to wear the accelerometer with a non-removable wrist strap to capture movement data continuously for seven complete days and nights (sampling rate of 80 Hz) at ages 5.5 and 8 years. To estimate the time spent in different movement behaviors, the raw acceleration data were processed using GGIR (version 2.0) in R software (39, 40). We set a minimum wear-time of ≥16 h/day (5-s epochs), which is a common approach in the literature (41, 42). The mean wear time at ages 5.5 and 8 years is 1440.12 (±1.55) and 1439.88 (±1.54) minutes per day, respectively. The vanHees2015 algorithm was applied to detect sustained inactivity and night sleep window (42, 43). GGIR derived 15-min periods of sustained inactivity outside the designated night sleep window is potentially indicative of napping time or rest (40). In Singapore and numerous other Asian countries and regions, napping constitutes a crucial component of the childcare centers and/or preschools daily schedule, with mandatory afternoon naps; moreover, most children continue to nap even beyond the preschool age (44–46). Therefore, we conducted a visual inspection of the data and noted that periods of at least 15 min of sustained inactivity during the afternoon on weekdays were consistently observed across the majority of children. These observations suggest that such periods are indicative of napping rather than sedentary behavior. Therefore, in this study, periods of sustained inactivity lasting at least 15 min within the non-sleep window were classified as napping time, in accordance with methodologies suggested in the literature (40). Total sleep time was calculated as the sum of time night sleep and naps. We applied Hildebrand et al. outpoints to categorize non-sleep time into inactivity (<35 m*g* of Euclidian Norm Minus One, 1 mg = 0.00981 m.s−^2^), LPA (35 to <200 m*g*) and MVPA (≥200 m*g*) (47, 48). Intuitively, inactivity time can be viewed as a proxy for SB time. However, it was not possible to determine posture using wrist-worn accelerometers, which is an essential element of SB definitions: any waking behavior while in a sitting, reclining or lying down posture characterized by energy expenditure ≤1.5 metabolic equivalent tasks (METs) (49, 50). Therefore, in this study, we used the term “inactivity” as a proxy for SB. Children with two valid weekdays and one valid weekend day were included in the analysis. The weighted averages of time spent on each activity across all valid days, where weekend days were weighted by 2/5 relative to the contribution of weekdays, were used in the analyses. In our study, the term ‘24-h movement behavior’ is used specifically to refer to accelerometer-measured data encompassing a full 24-h cycle, which includes PA, SB, and sleep. The term ‘movement behavior’ refers to any combination of these behaviors.

### Domain-specific PA and SB indicators

During the study visits at ages 5.5 and 8 years, parents reported time spent by their children on various activities during weekdays (school day) and weekend days, including (i) indoor active play; (ii) outdoor active play; (iii) participation in organized PA/sports and in active commuting (walking or cycling) to/from school; (iv) SB separated into screen-based SB such as viewing on TV, computers, and mobile devices (e.g., mobile phones, tablets), as well as screen-based games on electronic devices (e.g., PlayStation, Nintendo, Nintendo DS, and XBOX) while sitting or lying down; and (v) non-screen-based SB such as reading books and playing board/card games. The items in the questionnaire were mainly adapted from the Preschool-age Physical Activity Questionnaire (Pre-PAQ) and modified to reflect the Singapore context (51, 52). Time spent in each activity per day was calculated for each timepoint [(weekday × 5 + weekend day × 2)/7] and used as the exposure variables in the analysis. Total screen viewing time (SVT) per day was calculated by summing the times spent on TV, computer, mobile devices, and electronic games for each time point.

### Assessment of executive function and academic achievement

The NEuroPSYchology test, second edition (NEPSY-II), is a test battery that allows tailored assessments across six neuropsychological domains (53). The GUSTO study administered the Inhibition and Word List Interference tasks of the NEPSY-II to children aged 8.5 years. The Inhibition task consists of a Naming condition, interspersed with Inhibition and Switching conditions. The Inhibition and Switching errors were used as the Inhibition and Switching outcomes. The Word List Interference task asks children to hold in mind lists of up to 5 words while they are told new word lists as interference. The repetition and recall scaled scores were used as the Working Memory outcome. A latent variable of executive function (EF) was then derived by factor analysis, using z-scores of Inhibition, Switching, and Working Memory outcomes. The model indices and factor loadings are illustrated in Supplementary Figure S1.

The Wechsler Individual Achievement Test, Third Edition (WIAT-III) is a standardized and comprehensive test for assessing academic achievement (54). The GUSTO study used the WIAT-III to evaluate four academic domains, including spelling, oral reading rate, oral reading accuracy and numerical operations, among children at age 9 years. In the same way, a latent academic achievement variable was generated by factor analysis, using z-scores of the four academic domains. The model indices and factor loadings are illustrated in Supplementary Figure S2.

### Covariates

Maternal age at recruitment and maternal educational level at age 5 years visit were obtained as part of interviewer-administered questionnaires. Children’s dates of birth, sex, and ethnicity were ascertained from the hospital’s medical records. Weight (to the nearest gram) and height (to the nearest 0.1 cm) of children were measured at age 5 years using a weighing scale (SECA model 803) and a stadiometer (SECA model 213, Hamburg, Germany), respectively, and these measures were used to calculate body mass index (BMI, kg/m^2^).

### Statistical methods

In our study, accelerometer-measured 24-h movement behaviors and domain-specific PA and SB assessed at ages 5.5 and 8 years were used as exposure variables. Executive function and academic achievement assessed between ages 8.5 to 9 years served as outcome variables. This approach provides a unique opportunity to explore the prospective associations of early and later age’s movement behaviors with cognitive outcomes among school-aged children. We used chi-squared tests and two-sample t-tests to compare the categorical and continuous characteristics, respectively, of children included and excluded from this study. The accelerometer-measured 24 h-movement behavior compositions, including MVPA, LPA, inactivity, and total-sleep time, were analyzed using the compositional data analysis (CoDA) method (55–57). The CoDA models were executed using the Compositions version 2.0–4 package (58).

CoDA takes into account the fact that the proportions of time spent in different activities must add up to 100%, and allows researchers to account for the interdependency of time used in different activities. The isometric log-ratio (ilr) coordinate sets were constructed using a sequential binary partition, as described by Chastin et al., to express 24 h-movement behavior composition (57). For example, a set of three *ilr*-coordinates with MVPA, LPA, inactivity and sleep as the sequence of four behaviors in the composition were:

$ilr_{1}=\sqrt{\frac{3}{4}}\ln\left(\frac{MVPA}{\sqrt[{3}]{LPA*Inactivity*Sleep}}\right)$ ; $ilr_{2}=\sqrt{\frac{2}{3}}\ln\left(\frac{LPA}{\sqrt[{2}]{Inactivity*Sleep}}\right)$ ; $ilr_{3}=\sqrt{\frac{1}{2}}\ln\left(\frac{Inactivity}{Sleep}\right)$ . In the CoDA regression models, with the composition data as the exposure, a set of *ilr*-coordinates were included as predictors in the same regression model. The first *ilr*-coordinate (*ilr_1_*) expressed the relative time spent in first behavior in the sequence compared to the remaining three behaviors. Following Chastin et al., only *ilr_1_* was used to interpret the associations between the behaviors and the outcome. Thus, we constructed four sets of three *ilr*-coordinates for each timepoint by iterating the transformation, where each behavior (MVPA or LPA or inactivity or sleep) was the first behavior in the sequence (i.e., the numerator of ratio in *ilr_1_*). The prospective associations of accelerometer measured 24 h-movement behavior at age 5.5 and at age 8 years with executive functions and academic achievement were investigated using compositional multiple linear regression models. Model 1 was unadjusted, a set of *ilr_s_* of corresponding 24 h-movement behavior were used as exposures, and the results were interpreted based on *ilr_1_*, which accounts for the interdependency of time use in other movement behaviors. Model 2, was adjusted for potential covariates such as sex, ethnicity, maternal age, maternal education, and child BMI (in kg/m^2^), in addition to corresponding *ilr_s_*. Compositional isotemporal substitution method was used to estimate the changes in the executive function and academic achievement corresponding to the pair-wise reallocations of 5 to 60 min at 5-min increments between any two behaviors at their compositional mean, using the fitted regression models (i.e., Model 2).

The domain-specific PA and SB data at ages 5.5 and 8 years showed that almost half of the children did not engage in organized PA/sports, active commuting to/from school, and playing board/card games; therefore, each of these variables was dichotomized into two groups (Yes/No) instead of using continuous variables. Time spent in indoor and outdoor active play variables were skewed with outliers, and were categorized into low, medium and high based on tertiles. Time spent in total SVT, TV and handheld devices, and reading books were used as continuous variables in the analysis (h/day). Multiple linear regression models were used to assess the associations of domain-specific PA and SB at ages 5.5 and 8 years with executive function and academic achievement. Model 1 is the unadjusted model that includes only an exposure variable, while Model 2 is adjusted for potential confounders, including sex, ethnicity, maternal age, maternal education, and child BMI. In addition, adjusted models that investigated domain-specific PA included all the above-listed domain-specific PA variables, accelerometer-measured inactivity, and total sleep. Similarly, adjusted models that investigated domain-specific SB included total SVT (or TV and handheld devices time), playing board/card games and reading books, accelerometer-measured MVPA, and total sleep.

The sample sizes of the models using movement behaviors at 5.5 years and those using movement behaviors at 8 years were not consistent. To overcome this, we conducted sensitivity analyses using a subset of participants who had complete data on movement behaviors at both 5.5 and 8 years of age, as well as executive function data (*n* = 235) and academic achievement data (*n* = 216). All analyses were conducted in R version 4.1.1 (R Development Core Team, Vienna, Austria), and the statistical significance of associations was set at *p* < 0.05.

## Results

In the GUSTO cohort study, 748 children had at least one timepoint with valid 24 h-movement behavior data and domain-specific movement behaviors data. 432 of these children had executive function and academic achievement data, and they were included in the analysis (Figure 1). The included children were more likely to be of Malay ethnicity (27% vs. 20%) and less likely to be children of mothers with university-level education (33% vs. 45%), compared to excluded children (Table 1). Table 2 presents the description of 24 h-movement behaviors, domain-specific PA and domain-specific SB at ages 5.5 and 8 years, as well as the executive function and academic achievement of included children at 8.5 and 9 years, respectively.

**Table 1:** Comparison of characteristics between children included and excluded from this study in the GUSTO cohort.

	Included children (*N* = 432)	Excluded children (*N* = 316)	*P*-value^1^
Sex			0.274
Female	204 (47%)	162 (51%)	
Male	228 (53%)	154 (49%)	
Ethnicity			**0.013**
Chinese	251 (58%)	188 (59%)	
Malay	118 (27%)	62 (20%)	
Indian	63 (15%)	66 (21%)	
Maternal education			**0.005**
Before secondary	136 (32%)	73 (24%)	
Post-secondary	153 (35%)	95 (31%)	
University	143 (33%)	135 (45%)	
Missing data	0	13	
Maternal age at recruitment			0.996
<27 years	101 (23%)	74 (24%)	
27–33 years	177 (41%)	128 (41%)	
>33 years	154 (36%)	113 (35%)	
Missing data	0	1	
Body Mass Index of children (kg/m^2^), Mean (SD)			
At age 5 years	15.49 (1.85)	15.37 (2.28)	0.45
At age 8 years	16.42 (2.84)	16.16 (3.08)	0.24

SD, standard deviation. ^1^Pearson’s Chi-squared test for categorical variables; 2-sample *t*-test for continuous variables.

**Table 2:** Movement behaviors, executive function and academic performance of children at ages 5.5 and 8 years in the GUSTO cohort.

	Movement behaviors		Executive function/Academic performance
	At age 5.5 y (*n* = 314)	At age 8 y (*n* = 388)	At age 8.5 years/9 years
**Accelerometer measured 24 h movement behaviors (min/d), arithmetic mean (SD)**			
Moderate-to-vigorous physical activity	72 (24)	72 (26)	
Light physical activity	347 (48)	335 (54)	
Inactivity/sedentary behavior	487 (67)	510 (68)	
Total sleep duration	535 (42)	523 (45)	
**Domain-specific physical activity**			
Participate in organized physical activity (yes)	141 (45%)	174 (45%)	
**Levels of indoor active play time**			
Low	123 (39%)	144 (37%)	
Medium	103 (33%)	140 (36%)	
High	88 (28%)	104 (27%)	
**Levels of outdoor active play time**			
Low	117 (37%)	140 (36%)	
Medium	108 (34%)	122 (31%)	
High	89 (28%)	126 (32%)	
Active commuting time to school (yes)	181 (58%)	200 (52%)	
**Domain-specific inactivity/sedentary behavior**			
Screen viewing time, (min/d), Mean (SD)			
Total screen viewing	114 (87)	181 (128)	
Television viewing	57 (51)	88 (75)	
Handheld devices viewing	40 (45)	64 (71)	
Sitting and playing board/card games (yes)	104 (33)	137 (35)	
Reading books, Mean (SD)	25 (26)	34 (35)	
**Executive function score based on NEPSY-II at age 8.5 years**, Mean (SD)			0.01 (0.70)
**Academic achievement score based on WIAT-III at age 9 years**, Mean (SD)			0.03 (0.91)

SD, standard deviation; min/d, minutes per day; NEPSY, NEuroPSYchology test; WIAT, Wechsler Individual Achievement Test.

### Associations of accelerometer-measured 24 h-movement behavior with executive function and academic performance

Table 3 presents the associations of accelerometer-measured 24 h-movement behavior with executive function and academic achievement. We found no associations of 24 h-movement behaviors at ages 5.5 and 8 years with executive function and academic achievement. However, a higher amount of MVPA time relative to the remaining movement behaviors at age 5.5 years was associated with a lower mean academic achievement at age 9 years [Mean difference (95% confidence interval): −0.367 (−0.726, −0.009) z-score]. Relative to the remaining movement behaviors, time spent in LPA, inactivity or sleep at both ages was not associated with academic achievement at age 9 years.

**Table 3:** The associations of accelerometer measured movement behaviors at age 5.5 and 8 years with executive function at 8.5 years and academic achievement at age 9 years in the GUSTO cohort study.

	Executive function at age 8.5 years				Academic achievement at age 9 years			
	Model 1		Model 2		Model 1		Model 2	
	Mean difference (95% CI)	*p*-value	Mean difference (95% CI)	*p*-value	Mean difference (95% CI)	*p*-value	Mean difference (95% CI)	*p*-value
**24 h-MB at age 5.5 years**	*n* = 270	0.724	*n* = 270	0.758	*n* = 251	0.184	*n* = 251	0.147
MVPA relative to remaining behaviors	−0.145 (−0.414, 0.124)	0.290	−0.094 (−0.366, 0.178)	0.497	**−0.368 (−0.735, −0.001)**	**0.049**	**−0.367 (−0.726, −0.009)**	**0.044**
LPA relative to remaining behaviors	0.054 (−0.510, 0.618)	0.850	−0.074 (−0.632, 0.483)	0.793	0.228 (−0.589, 1.046)	0.582	0.08 (−0.700, 0.860)	0.839
Inactivity/SB relative to remaining behaviors	−0.067 (−0.547, 0.413)	0.784	0.055 (−0.420, 0.531)	0.819	−0.458 (−1.193, 0.277)	0.221	−0.291 (−0.977, 0.394)	0.404
Sleep relative to remaining behaviors	0.158 (−0.511, 0.826)	0.643	0.113 (−0.541, 0.767)	0.734	0.598 (−0.458, 1.653)	0.266	0.578 (−0.410, 1.567)	0.250
**24 h-MB at age 8 years**	*n* = 338	0.468	*n* = 338	0.682	*n* = 315	0.088	*n* = 315	0.267
MVPA relative to remaining behaviors	−0.195 (−0.442, 0.053)	0.123	−0.097 (−0.354, 0.160)	0.459	−0.285 (−0.617, 0.046)	0.092	−0.282 (−0.616, 0.052)	0.098
LPA relative to remaining behaviors	0.237 (−0.244, 0.717)	0.333	0.013 (−0.454, 0.479)	0.958	0.41 (−0.219, 1.039)	0.201	0.125 (−0.476, 0.725)	0.683
Inactivity/SB relative to remaining behaviors	−0.155 (−0.639, 0.328)	0.528	0.162 (−0.313, 0.638)	0.503	**−0.697 (−1.331, −0.063)**	**0.031**	−0.374 (−0.978, 0.229)	0.223
Sleep relative to remaining behaviors	0.113 (−0.510, 0.737)	0.721	−0.078 (−0.678, 0.522)	0.799	0.572 (−0.243, 1.388)	0.168	0.532 (−0.236, 1.300)	0.174

24 h-MB, 24 h movement behaviors; LPA, light physical activity; MVPA, moderate-to-vigorous physical activity; CI, confidence interval. Model 1: adjusted for ilr2 and ilr3 (isometric log-ratio of remaining 24 h-MB). Model 2: adjusted for ilr2, ilr3, sex, ethnicity, BMI at age 5 or 8 years and maternal age and education. Significant associations are shown in bold.

Applying the compositional isotemporal substitution method to the fitted models (i.e., Model 2), reallocation between the amount of time spent in MVPA and sleep at age 5.5 years showed significant changes in academic achievement (Figure 2A), while other pairs of reallocations were not significant (Supplementary Figures S3, S4). Reallocation of 5 to 60 min/day from MVPA to sleep at age 5.5 years was associated with a significant increase in mean academic achievement. For instance, reallocating 30 min from MVPA to sleep at age 5.5 resulted in a 0.214 (0.023, 0.404) increase in academic achievement z-score. Notably, the expected decrease in academic achievement z-score was relatively lower [−0.145 (−0.272, −0.0186)] when the same amount of time was reallocated from sleep to MVPA at age 5.5 years. Similar changes were observed when reallocating MVPA and sleep time at age 8 years but they were not significantly associated with academic achievement [0.170 (−0.010, 0.350) and − 0.117 (−0.236, 0.002) z-scores] (Figure 2B).

**Figure 2a:**
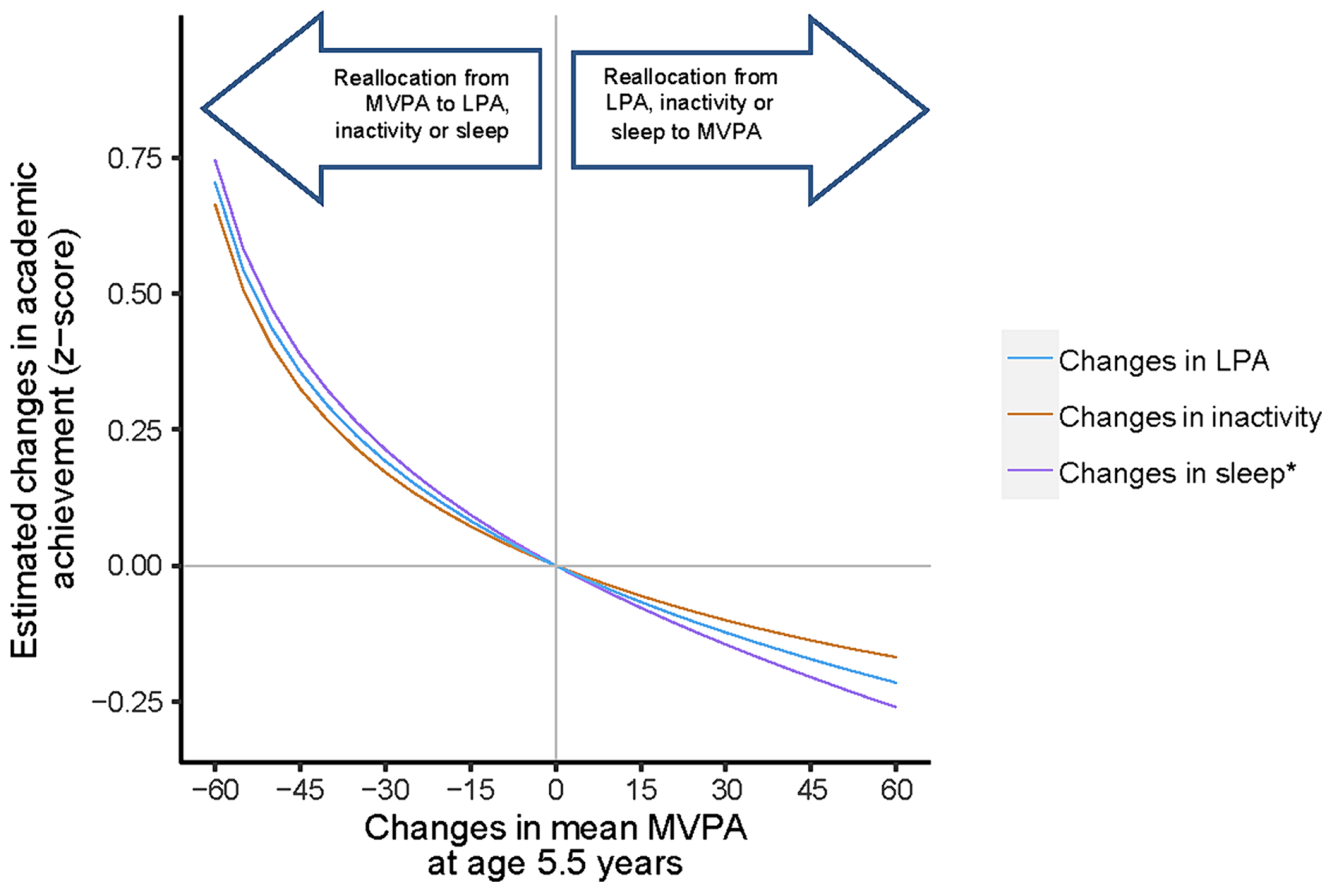
Estimated changes in the academic achievement scores associated with hypothetical reallocation of time from MVPA to each remaining behaviour at age 5.5 years in the GUSTO study. MVPA, moderate-to-vigorous physical activity; LPA, Light physical activity; MB, movement behaviour. Results were adjusted for sex, ethnicity, BMI at age 5 years and maternal age and education. Baseline compositional mean minutes of MVPA, LPA, inactivity and sleep are 68, 343, 487, 542 per day at age 5.5 years, respectively. *Corresponding change in the academic performance was statistically significant.

**Figure 2b:**
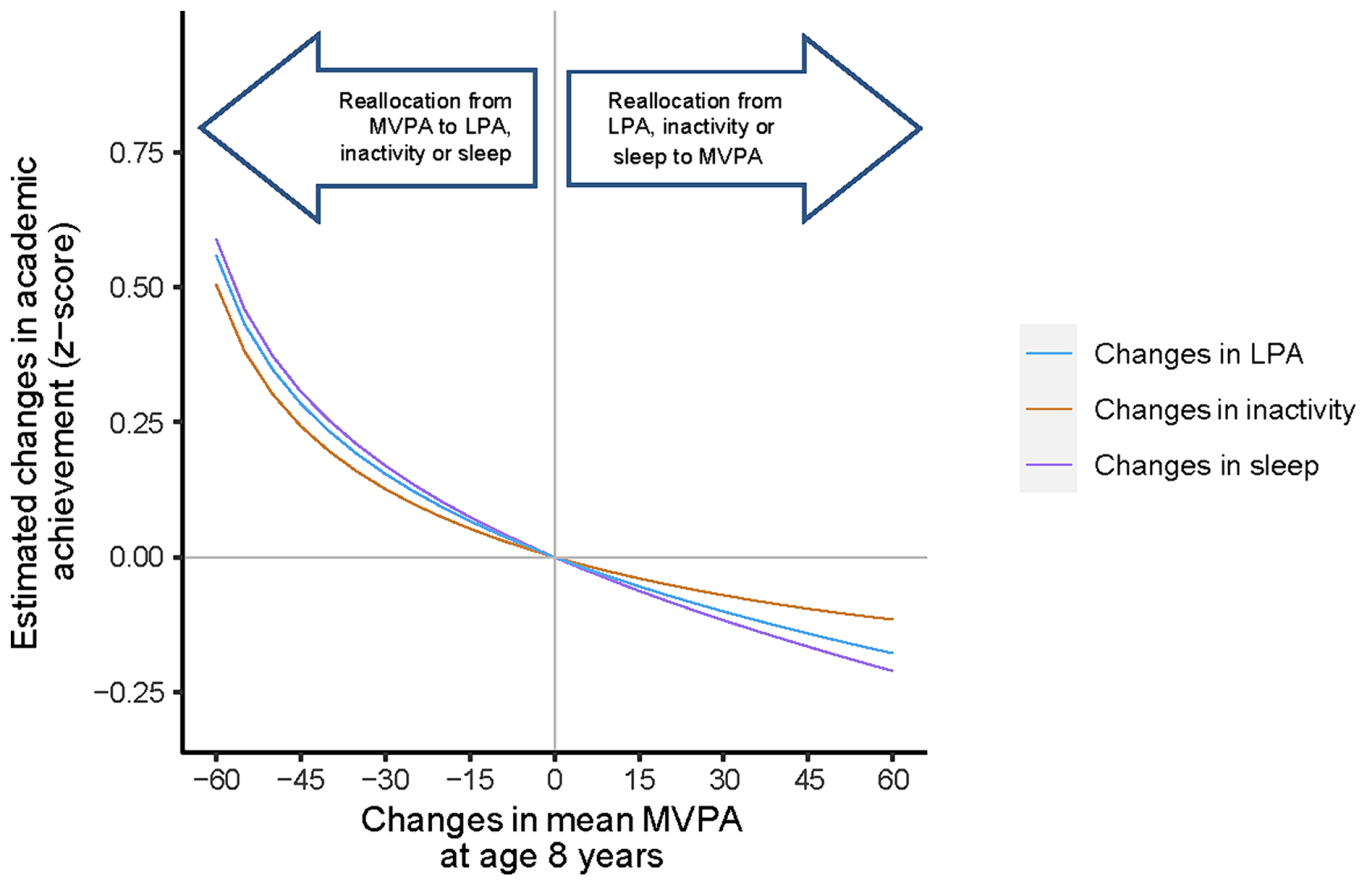
Estimated changes in the academic achievement scores associated with hypothetical reallocation of time from MVPA to each remaining behaviour at age 8 years in the GUSTO study. MVPA, moderate-to-vigorous physical activity; LPA, Light physical activity; MB, movement behaviour. Results were adjusted for sex, ethnicity, BMI at age 8 years and maternal age and education. Baseline compositional mean minutes of MVPA, LPA, inactivity and sleep are 67, 336, 509, 527 per day at age 8 years, respectively. *Corresponding change in the academic performance was statistically significant.

### Associations of domain-specific PA with executive function and academic achievement

From Table 4 we observed that children who participated in organized PA/sports at ages 5.5 and 8 years had a higher mean executive function [0.192 (0.026, 0.359) and 0.221 (0.069, 0.373) z-scores, respectively] at age 8.5 years. And children who participated in organized PA/sports at age 8 years had higher mean academic achievement [0.239 (0.036, 0.441) z-scores] at age 9 years. Although organized PA/sports at ages 5.5 did not show a significant association with academic achievement, it displayed a consistent pattern [0.201 (−0.031, 0.433) z-scores]. Indoor and outdoor active play at age 5.5 was not associated with executive function and academic achievement. However, compared to children who engaged in low levels of outdoor active play at age 8 years, those who engaged in medium and/or high levels had higher mean executive function [0.201 (0.022, 0.380) and 0.211 (0.023, 0.399) z-scores] and higher mean academic achievement [0.273 (0.039, 0.507) and 0.204 (−0.040, 0.448) z-scores]. Conversely, compared to children who engaged in low levels of indoor active play at age 8 years, those who engaged in high levels had lower mean executive function [−0.266 (−0.455, −0.076) z-score] and academic achievement [−0.250 (−0.500, 0.001) z-score]. Engagement in active commuting to/from school at age 5.5 and 8 years were not significantly associated with executive function and academic achievement.

**Table 4:** Prospective associations of parent/caregiver reported domain-specific physical activity at age 5.5 and 8 years with executive function at age 8.5 years and academic achievement scores at age 9 years in the GUSTO cohort study.

	Executive function at age 8.5 years				Academic achievement at age 9 years			
	Model 1		Model 2		Model 1		Model 2	
	Mean difference (95% CI)	*p*-value	Mean difference (95% CI)	*p*-value	Mean difference (95% CI)	*p*-value	Mean difference (95% CI)	*p*-value
**Physical activity at age 5.5 years**	*n* = 270		*n* = 270		*n* = 251		*n* = 251	
Organized physical activity (Yes)	**0.264 (0.112, 0.416)**	**<0.001**	**0.192 (0.026, 0.359)**	**0.024**	**0.383 (0.165, 0.601)**	**0.001**	0.201 (−0.031, 0.433)	0.090
Levels of outdoor active play time		0.807		0.802		0.397		0.716
Low	ref				ref			
Medium	−0.045 (−0.226, 0.136)	0.624	−0.060 (−0.239, 0.120)	0.515	0.112 (−0.153, 0.378)	0.406	0.104 (−0.152, 0.359)	0.425
High	−0.06 (−0.256, 0.136)	0.545	−0.019 (−0.225, 0.188)	0.858	−0.077 (−0.354, 0.200)	0.585	0.035 (−0.247, 0.316)	0.809
Levels of indoor active play time		0.981		0.992		0.612		0.709
Low	ref				ref			
Medium	−0.013 (−0.197, 0.171)	0.887	0.000 (−0.183, 0.183)	0.999	−0.091 (−0.356, 0.173)	0.497	−0.073 (−0.325, 0.179)	0.569
High	−0.018 (−0.209, 0.174)	0.857	0.011 (−0.191, 0.214)	0.912	−0.132 (−0.406, 0.143)	0.346	−0.113 (−0.392, 0.166)	0.425
Active commuting to school (Yes)	−0.048 (−0.204, 0.109)	0.549	0.013 (−0.146, 0.173)	0.869	−0.16 (−0.385, 0.064)	0.161	−0.024 (−0.248, 0.200)	0.833
**Physical activity at age 8 years**	*n* = 338		*n* = 338		*n* = 315		*n* = 315	
Organized physical activity (Yes)	**0.365 (0.220, 0.510)**	**<0.001**	**0.221 (0.069, 0.373)**	**0.004**	**0.45 (0.254, 0.645)**	**<0.001**	**0.239 (0.036, 0.441)**	**0.021**
Levels of outdoor active play time		**0.017**		**0.043**		**0.006**		0.064
Low	ref		ref		ref		ref	
Medium	**0.250 (0.070, 0.430)**	**0.007**	**0.201 (0.022, 0.380)**	**0.027**	**0.395 (0.152, 0.638)**	**0.002**	**0.273 (0.039, 0.507)**	**0.022**
High	**0.188 (0.008, 0.368)**	**0.041**	**0.211 (0.023, 0.399)**	**0.028**	**0.240 (0.003, 0.478)**	**0.047**	0.204 (−0.040, 0.448)	0.100
Levels of indoor active play time		**0.021**		**0.015**		0.063		**0.033**
Low	ref		ref		ref		ref	
Medium	0.013 (−0.160, 0.187)	0.881	−0.052 (−0.222, 0.117)	0.543	0.099 (−0.134, 0.332)	0.403	0.055 (−0.169, 0.279)	0.629
High	**−0.229 (−0.418, −0.040)**	**0.018**	**−0.266 (−0.455, −0.076)**	**0.006**	−0.204 (−0.457, 0.049)	0.113	−0.250 (−0.500, 0.001)	0.051
Active commuting to school (Yes)	−0.006 (−0.155, 0.144)	0.940	0.032 (−0.109, 0.173)	0.660	−0.095 (−0.296, 0.106)	0.354	−0.029 (−0.214, 0.157)	0.763

MB, movement behaviors; CI, confidence interval. Model 1: unadjusted model. Model 2: adjusted for child sex, ethnicity, BMI at age 5 or 8 years and maternal age and education + accelerometer-measured inactivity and sleep. Organized physical activity, indoor and outdoor active play and active commuting were mutually adjusted. Significant associations are shown in bold.

### Associations of domain-specific SB with executive function and academic achievement

Total SVT, TV and handheld screen device viewing time at ages 5.5 and 8 years were not associated with executive function (Table 5). However, SVT showed inverse directions of associations with academic achievement; higher amount of time spent in total SVT and TV at age 5.5 years [−0.071 (−0.149, 0.006) and − 0.131 (−0.262, 0.000) z-scores] and at age 8 years [−0.062 (−0.106, −0.018) and-0.091 (−0.167, −0.015) z-scores] showed lower mean academic achievement at age 9 years. Handheld devices at both 5.5 and 8 years did not show a significant association with academic achievement [−0.058 (−0.203, 0.088) and-0.071 (−0.150, 0.009) z-scores]. The amount of time spent reading books at age 8 years was positively associated with an increase of executive function 0.134 (0.009, 0.259) z-score and academic achievement 0.302 (0.132, 0.472) z-score, while no statistically significant associations for reading books at age 5.5 years was observed. We also did not find associations of playing board/card games at ages 5.5 and 8 years with executive function and academic achievement.

**Table 5:** Prospective associations of parents/caregivers reported domain-specific sedentary behavior at ages 5.5 and 8 years with executive function at age 8.5 years and academic achievement scores at age 9 years in the GUSTO cohort study.

	Executive function at age 8.5 years				Academic achievement at age 9 years			
	Model 1		Model 2		Model 1		Model 2	
	Mean difference (95% CI)	*p*-value	Mean difference (95% CI)	*p*-value	Mean difference (95% CI)	*p*-value	Mean difference (95% CI)	*p*-value
**Sedentary behavior at age 5.5 years**	*n* = 270		*n* = 270		*n* = 251		*n* = 251	
Total SVT, h/day	−0.012 (−0.065, 0.042)	0.664	0.004 (−0.051, 0.059)	0.884	**−0.098 (−0.175, −0.020)**	**0.014**	−0.071 (−0.149, 0.006)	0.072
Television time, h/day	0.004 (−0.086, 0.093)	0.934	0.012 (−0.080, 0.105)	0.794	**−0.154 (−0.289, −0.020)**	**0.025**	−0.131 (−0.262, 0.000)	0.051
Handheld devices time, h/day	−0.043 (−0.147, 0.061)	0.418	−0.011 (−0.119, 0.096)	0.835	−0.124 (−0.272, 0.023)	0.098	−0.058 (−0.203, 0.088)	0.435
Playing board/card games (yes)	0.003 (−0.161, 0.168)	0.972	−0.034 (−0.200, 0.131)	0.683	0.157 (−0.078, 0.393)	0.189	0.082 (−0.143, 0.307)	0.473
Reading books, h/day	0.142 (−0.027, 0.311)	0.099	0.142 (−0.026, 0.309)	0.097	**0.303 (0.020, 0.587)**	**0.036**	0.187 (−0.087, 0.460)	0.180
**Sedentary behavior at age 8 years**	*n* = 338		*n* = 338		*n* = 315		*n* = 315	
Total SVT, h/day	−0.029 (−0.064, 0.005)	0.097	0.003 (−0.031, 0.038)	0.858	**−0.095 (−0.141, −0.049)**	**<0.001**	**−0.062 (−0.106, −0.018)**	**0.006**
Television time, h/day	−0.057 (−0.115, 0.001)	0.056	−0.015 (−0.072, 0.042)	0.603	**−0.166 (−0.245, −0.087)**	**<0.001**	**−0.091 (−0.167, −0.015)**	**0.019**
Handheld devices time, h/day	−0.008 (−0.072, 0.055)	0.797	0.043 (−0.019, 0.105)	0.174	**−0.127 (−0.211, −0.043)**	**0.003**	−0.071 (−0.150, 0.009)	0.081
Playing board/card games (yes)	0.140 (−0.014, 0.294)	0.076	0.095 (−0.054, 0.245)	0.211	0.015 (−0.195, 0.226)	0.886	−0.068 (−0.262, 0.126)	0.493
Reading books, h/day	**0.221 (0.094, 0.348)**	**0.001**	**0.134 (0.009, 0.259)**	**0.036**	**0.408 (0.228, 0.589)**	**<0.001**	**0.302 (0.132, 0.472)**	**<0.001**

MB, movement behaviors; CI, confidence interval. Model 1: unadjusted. Model 2: adjusted for child sex, ethnicity, BMI at age 5 or 8 years and maternal age and education + accelerometer-measured moderate-to-vigorous physical activity and sleep. Total screen viewing time or television and handheld devices time, playing board/card games and reading books were mutually adjusted. Significant associations are shown in bold.

The findings in the sensitivity analyses remained largely consistent despite some modest changes in the magnitude of associations (Supplementary Tables S1–S3).

## Discussion

This is the first cohort study to investigate the prospective associations of the full spectrum of accelerometer-measured 24 h-movement behavior (LPA, MVPA, inactivity and sleep), as well as domain-specific PA and SB, with executive function and academic achievement among children. In our study, accelerometer-measured overall 24 h-movement behaviors at ages 5.5 and 8 years were not associated with executive function at age 8.5 years or academic achievement at age 9 years. However, reallocating time from MVPA to sleep was associated with higher academic achievement. Moreover, the relationships between PA and SB with investigated outcomes differed considerably across behavioral domains. In particular, participation in organized PA/sports, higher levels of outdoor active play and longer reading time were associated with favorable executive function and academic achievement. On the other hand, higher levels of indoor active play and more screen time were associated with unfavorable executive function and/or academic achievement.

Past reviews and meta-analyses suggested that PA and sleep are positively associated with executive function and/or academic achievement in school-age children aged 5–12 years (9, 30). Associations of SB with executive function or academic achievement were inconclusive in the literature, and previous studies have largely reported negative or no associations with cognitive outcomes (18–20). However, most of the evidence to date has been based on cross-sectional studies and only used parent-reported data on MVPA, sleep and screen-based SB, not considering LPA, total PA or total SB (9, 10, 18, 29, 30). Moreover, the interdependency of various movement behaviors was not accounted for in previous studies. We bridged this gap by investigating the prospective associations of 24 h-movement behaviors with executive function. We found no evidence for associations of accelerometer-measured LPA, MVPA, inactivity and sleep with executive function after accounting for the interdependency of time spent in these behaviors among school-aged children. Bazerra et al. previously conducted a cross-sectional study among pre-school age (3–5 years) children; the study reported that the reallocation of 5–20 min/day from LPA to MVPA improved executive function, whereas reallocating the same amount of time from MVPA to sleep or from sleep to LPA decreased executive function (37). These contradictory findings may be due to reverse causation or differences in several influencing factors, such as differences in children’s age and other socio-demographic and environmental factors (59, 60). In our study of Singaporean children, we did not find associations of 24 h-movement behaviors at age 5.5 and 8 years with academic achievement at age 9 years. However, a higher relative amount of time spent in MVPA at age 5.5 years may be negatively associated with academic achievement and reallocating time from MVPA to sleep could have a positive impact on academic achievement. A meta-analysis suggests that sleep duration is associated with improved cognitive outcomes among children (30). We previously reported, though, that a higher proportion of 5.5 year-old children (~85%) in Singapore were sleeping less than the recommended 9 h per night (44). This lack of sufficient sleep may explain the positive impact of higher sleep duration in the present study. Extracurricular academic enrichment classes are an activity in which children in Singapore and many Asian countries frequently engage (61). Taken together, the potential negative association between MVPA and academic achievement may be explained by displacement of sleep time or academic activity time; this hypothesis warrants further investigation. In the present study, the amount of time spent in LPA and total SB (inactivity) did not show any associations with academic achievement. Recent systematic reviews have reported varying findings, indicating either positive or no associations between LPA and cognitive outcomes (62), and either negative or no associations between SB and cognitive outcomes (18), among children and adolescents. Given the limitations of previous studies, our findings highlight the need for further prospective studies to strengthen the evidence base.

Throughout each day, PA and SB are accumulated in different contexts and by engaging in diverse domains of activities. These activities contribute toward total PA and total SB, but specific activities or activities conducted in specific contexts may exhibit variable effects on cognitive and learning outcomes of children (15–17, 19, 23). Thus far, the available evidence is primarily based on investigations of individual domains/contexts of PA and SB, without consideration of the time spent on other movement behaviors (15–17, 19, 23). Moreover, previous studies have not reported the overall and domain/context-specific associations in the same group of participants. Our findings present a complex picture across a range of specific types of PA and SB. We found that participating in organized PA/sports was consistently associated with higher executive function and academic achievement. Our result is supported by a systematic review that also shows a positive association between engaging in sports and cognitive function (15). One of the possible explanations for the observed associations is that organized PA/sports are frequently group activities that may provide an equilibrium between individual and group demands for children, challenge their inhibition and working memory, train them to shift their attention efficiently, and consequently improve their academic performance (15, 63). We also found that a higher level of active outdoor play at age 8 years was favorably associated with executive function and academic achievement, whereas a higher level of active indoor play was detrimental. These findings are supported by an experimental study among pre-school children demonstrating positive associations of outdoor play but negative associations of indoor play with executive function (64). The reasons for these context-specific associations are not entirely clear. A possible explanation is that exposure to natural environments during outdoor play has been linked to improvements in attention, memory, problem-solving skills, sensory stimulation, spatial awareness, social interaction, and hands-on learning experiences across a variety of activities, all essential for cognitive development (64–68). Moreover, outdoor play is known to provide psychological benefits such as stress reduction and mood improvement, which in turn enhance executive function compared to indoor play (68, 69). Further research is warranted to explore the associations of organized PA/sports and outdoor active play with cognitive outcomes.

Our study did not show evidence of associations between active commuting to and from school and outcomes of interest. Consistent with our findings on active transportation, a recent systematic review and meta-analysis also reported no evidence for associations between active commuting and executive function or academic achievement in school-aged children (16). However, the findings of associations varied across studies (16, 70). It is possible that active transportation in certain countries does not involve a high level of cognitive engagement, while this may be different in others. For example, in developed countries like Singapore, with pedestrian-friendly infrastructure and bike lanes, active transportation to school may not require a high level of cognitive engagement as the routes are safe and straightforward. The proximity of kindergarten and primary schools to homes in Singapore, or shorter distances to public transport, may also have contributed to the lack of associations of active transport to school with executive function and academic achievement. The inconsistency of findings warrants further investigation.

Previous research has predominantly focused on the associations of screen viewing, particularly television viewing, within the spectrum of SB in children (19, 22, 71). However, comprehensive analyses of other SB domains, such as reading books and sitting and playing board/card games, are less common in the literature. In our study, domain-specific analyses reveal that SVT, particularly TV viewing, is negatively associated with academic achievement, though it shows no significant associations with executive function. This is partly supported by a systematic review, which found that TV viewing negatively associated with academic performance among children and adolescents aged 4 to 18 years, whereas total SVT was not associated with academic performance (22). Similar to our study, a recent meta-analysis reported that SVT was not associated with executive function in children under 6 years of age (71). In contrast to screen viewing, our findings indicate that reading time, which has been less emphasized in SB research, positively correlates with both executive function and academic achievement. These results align with a systematic review indicating that while TV viewing has been associated with poorer cognitive development outcomes, reading was associated with improved cognitive development in children under 5 years old (19). They also appear to reflect the time displacement hypothesis, which posits that the time children spend in academic activities, such as reading, writing or listening to stories, replaces the time they spend on screen use (72). Moreover, reading demands cognitive efforts that promote intrinsic motivation and problem-solving abilities (72, 73), whereas passive screen viewing, such as TV viewing, may alter reward pathways (23). No previous studies have investigated the associations of sitting and playing board/card games with executive function and academic achievement. We found no evidence of an association, but the complex relationships between different types of activities and cognitive outcomes in children warrant further studies exploring a wider range of activities.

Our result suggests that the associations of specific domains of PA and SB on cognitive outcome becomes more pronounced as children grow. The patterns of their domain-specific PA and SB tend to become more established among older children (74, 75), and the cognitive outcomes assessed at age 9 are temporally closer to the behaviors observed at age 8. This proximity in time may explain the stronger associations observed at age 8 years compared to those at age 5.5 years. Moreover, we observed a significant negative association between accelerometer-measured MVPA at age 5.5 years and academic achievement, although MVPA at year 8 showed a similar direction of association, it was not statistically significant. These inconsistent findings regarding the association of MVPA may be attributed to variations in how domain-specific PA influences cognitive outcomes at different ages. Upon reflection of domain-specific findings in our study, we hypothesize that the negative associations of accelerometer-measured MVPA at age 5.5 years with academic achievement could be attributed to a higher proportion of MVPA consisting of indoor active play or unstructured PA. These types of activities often lack the cognitive challenges and structured skill development that are integral to organized sports and activities typically conducted outdoors (15, 63, 69), which were found to be positively associated with both executive function and academic achievement in our study. The stronger and contrasting directions of domain-specific PA associations observed at age 8 years could account for the non-significant associations of accelerometer-measured MVPA at this age. This nuanced perspective enhances our understanding of how specific domains of PA differentially contribute to cognitive outcomes. Further research involving a larger sample size could enable a detailed analysis of the domains of PA and SB, providing clearer insights into the mechanisms of the observed associations.

Overall, this is the first prospective study to utilize comprehensive accelerometer-measured 24 h-movement behaviors, a wide range of parent-reported domain-specific PA and SB data, and objectively measured cognitive outcomes. Our study highlights the potential consequences of reallocating time between behaviors, but also the importance of considering the impact of specific activities. For instance, promoting organized PA/sports and outdoor active play while ensuring adequate sleep could help to improve cognitive outcomes. Conversely, a higher amount of indoor active play time may not be beneficial for cognitive development. Similar to PA, our study demonstrates the variable associations of different domains of SB on cognitive outcomes, underscoring the importance of spending more time on activities such as reading and reducing screen usage. These observations may explain the lack of an overall association between total time spent being inactive (or SB) and cognitive outcomes. Taken together, our study contributes significantly to the current literature and provides insights into potential opportunities for future research and the development of strategies to improve executive function and academic achievement in children. Our study also strengthens the evidence base for current 24 h-movement behavior guidelines in school-aged children, which emphasize promoting regular MVPA, limiting SVT, and having adequate sleep for optimal health and development (76, 77). Future prospective studies using comprehensive and high-quality measures are warranted to investigate these complex relationships and interdependency of movement behaviors. Attention should be paid to activities that require social skills, such as organized PA/sports and outdoor play, as well as sedentary activities that actively engage the mind, such as reading. Further studies investigating the trade-offs between time spent in different activities could provide valuable insights to develop appropriate health promotion strategies.

One of the major limitations of our study, as noted in the methods, is that data on movement behaviors and cognitive outcomes were collected at different, non-coincident time points. This separation posed challenges in accounting for baseline cognitive outcomes when assessing longitudinal associations. Despite this, the study provides valuable insights into the potential long-term and short-term associations of early-life movement behaviors on later cognitive outcomes, highlighting the need for more synchronized data collection in future research. We used data on domain-specific PA and SB reported by parents/caregivers, rather than data obtained through objective measurements. This approach may have introduced reporting errors. Residual confounding due to other unmeasured factors, such as physical fitness, mental health, and school and household environmental factors, cannot be excluded. For the measurement of 24-h movement behaviors, wrist-worn accelerometers were selected due to their demonstrated high compliance rate among participants (50). However, it is crucial to acknowledge specific limitations associated with this placement. Notably, wrist-worn accelerometers have limitations in distinguishing between periods of sedentary behavior-related inactivity and nap time (39, 40, 48, 50). Given these distinctions, further research is necessary to validate our findings. Finally, the GUSTO cohort is not representative of the Singapore population (38), which might affect the generalizability of our results.

In conclusion, our findings provide insights into the complex relationships between movement behaviors and cognitive outcomes in children, emphasizing the importance of considering the domains and contexts of PA and SB. While overall accelerometer measured 24 h-movement behaviors were not associated with executive function and academic achievement later during childhood, a higher relative amount of time spent in MVPA may be negatively associated with academic achievement, especially if it displaces Singaporean children’s already limited sleep duration. Moreover, specific types of activity appear to matter for children’s cognitive development. While organized PA, outdoor play, and reading books were favorably associated with cognitive outcomes, no associations and even the opposite were observed for active commuting, indoor play and screen-viewing. These findings underscore the importance of elucidating the varying levels of cognitive and social engagement required by different domains and contexts of PA and SB, and how they may relate to children’s cognitive development. Further prospective studies with larger sample sizes and an exploration of the trade-offs between different domains and contexts of PA and SB would be valuable in informing the development of future guidelines and effective health promotion strategies.

## Data availability statement

The raw data supporting the conclusions of this article will be made available by the authors, without undue reservation.

## Ethics statement

The studies involving humans were approved by the study received ethical approval from the National Healthcare Group Domain Specific Review Board and the SingHealth Centralized Institutional Review Board. The studies were conducted in accordance with the local legislation and institutional requirements. Written informed consent for participation in this study was provided by the participants’ legal guardians/next of kin.

## Author contributions

NP: Conceptualization, Data curation, Formal analysis, Investigation, Methodology, Supervision, Visualization, Writing – original draft, Writing – review & editing. JB: Conceptualization, Formal analysis, Funding acquisition, Methodology, Resources, Supervision, Writing – review & editing. SYT: Writing – review & editing, Formal analysis, Investigation, Validation. AC: Conceptualization, Validation, Writing – review & editing. CG: Data curation, Formal analysis, Visualization, Writing – review & editing. SLT: Data curation, Formal analysis, Validation, Visualization, Writing – review & editing. LS: Funding acquisition, Project administration, Resources, Writing – review & editing, Validation. YSC: Conceptualization, Funding acquisition, Project administration, Resources, Writing – review & editing, Validation. KHT: Conceptualization, Project administration, Resources, Writing – review & editing. S-YC: Conceptualization, Funding acquisition, Project administration, Resources, Writing – review & editing. FY: Funding acquisition, Project administration, Resources, Writing – review & editing. KMG: Funding acquisition, Resources, Supervision, Writing – review & editing. YSL: Writing – review & editing, Project administration. MM: Project administration, Writing – review & editing. JE: Project administration, Supervision, Writing – review & editing. CST: Data curation, Formal analysis, Supervision, Writing – review & editing. EL: Data curation, Formal analysis, Project administration, Supervision, Writing – review & editing. FM-R: Conceptualization, Data curation, Formal analysis, Investigation, Methodology, Project administration, Resources, Supervision, Validation, Visualization, Writing – review & editing.
