# Supplementary material for: The prospective associations of 24-hour movement behaviors and domain-specific activities with executive function and academic achievement among school-aged children in Singapore
Source: Front Public Health. 2024 Sep 4;12:1412634. doi: 10.3389/fpubh.2024.1412634 (PMC11409845; doi:10.3389/fpubh.2024.1412634)
Supplement: Supplementary file 1 [file Data_Sheet_1.docx]

Supplementary Figure 1: Latent variable for executive function

# Multiplied by minus 1 in order to generate positive factor loading.

Covariate terms were included between repetition and recall score.

Model indices of Confirmatory Factor Analysis: chi-square p-value = 0.134; Comparative Fit Index (CFI) = 0.991; Tucker-Lewis Index (TLI) = 0.945; Root Mean Square Error of approximation (RMSEA) = 0.055; Standardized Root Mean Square Residual (SRMR) = 0.016

*statistically significant

Supplementary Figure 2: Latent variable for academic achievement

Covariate terms were included between oral reading accuracy, fluency and reading rate.

Model indices of Confirmatory Factor Analysis: chi-square p-value = 0.414; Comparative Fit Index (CFI) = 1.000; Tucker-Lewis Index (TLI) = 1.002; Root Mean Square Error of approximation (RMSEA) = 0.000; Standardized Root Mean Square Residual (SRMR) = 0.005

*statistically significant

Supplementary Table 1: The associations of accelerometer measured movement behaviours at age 5.5 and 8 years with executive function at 8.5 years and academic achievement at age 9 years in the GUSTO cohort study (Sensitivity analysis based on complete case)

|  | **Executive function at age 8.5 years** | | **Academic achievement at age 9 years** | |
| --- | --- | --- | --- | --- |
|  | **Model 2** | | **Model 2** | |
|  | Mean difference (95% CI)  n=235 | p-value | Mean difference (95% CI)  n=216 | p-value |
| **24h-MB at age 5.5 years** |  | 0.683 |  | 0.246 |
| MVPA relative to remaining behaviours | -0.036 (-0.334, 0.262) | 0.811 | -0.329 (-0.729, 0.071) | 0.107 |
| LPA relative to remaining behaviours | -0.247 (-0.843, 0.348) | 0.414 | 0.043 (-0.804, 0.890) | 0.921 |
| Inactivity/SB relative to remaining behaviours | 0.042 (-0.463, 0.546) | 0.871 | -0.423 (-1.172, 0.326) | 0.266 |
| Sleep relative to remaining behaviours | 0.242 (-0.458, 0.942) | 0.496 | 0.709 (-0.380, 1.799) | 0.201 |
| **24h-MB at age 8 years** |  | 0.388 |  | 0.331 |
| MVPA relative to remaining behaviours | -0.227 (-0.513, 0.058) | 0.118 | -0.358 (-0.755, 0.039) | 0.077 |
| LPA relative to remaining behaviours | 0.287 (-0.234, 0.807) | 0.279 | 0.212 (-0.515, 0.939) | 0.566 |
| Inactivity/SB relative to remaining behaviours | 0.089 (-0.440, 0.617) | 0.741 | -0.254 (-0.974, 0.465) | 0.487 |
| Sleep relative to remaining behaviours | -0.148 (-0.814, 0.519) | 0.662 | 0.401 (-0.519, 1.320) | 0.391 |
| 24h-MB, 24h movement behaviours; LPA, light physical activity; MVPA, moderate-to-vigorous physical activity; CI, confidence interval  Model 2: adjusted for ilr2, ilr3, sex, ethnicity, BMI at age 5 or 8 years and maternal age and education  Significant associations are shown in bold | | | | |

Supplementary Table 2: Prospective associations of parent/caregiver reported domain-specific physical activity at age 5.5 and 8 years with executive function at age 8.5 years and academic achievement scores at age 9 years in the GUSTO cohort study (Sensitivity analysis based on complete case)

|  | **Executive function at age 8.5 years** | | **Academic achievement at age 9 years** | |
| --- | --- | --- | --- | --- |
|  | **Model 2** | | **Model 2** | |
|  | Mean difference (95% CI) | p-value | Mean difference (95% CI) | p-value |
| **At age 5.5 years** | n=235 |  | 216 |  |
| Organized physical activity (Yes) | **0.179 (0.001, 0.356)** | **0.049** | 0.241 (-0.011, 0.492) | 0.060 |
| Levels of outdoor active play time |  | **0.795** |  | 0.993 |
| Low | ref |  | ref |  |
| Medium | -0.060 (-0.251, 0.131) | 0.537 | -0.010 (-0.264, 0.283) | 0.943 |
| High | -0.004 (-0.223, 0.215) | 0.973 | -0.007 (-0.312, 0.297) | 0.962 |
| Levels of indoor active play time |  | 0.957 |  | 0.465 |
| Low | ref |  | ref |  |
| Medium | 0.001 (-0.194, 0.196) | 0.991 | -0.141 (-0.409, 0.126) | 0.299 |
| High | 0.029 (-0.189, 0.247) | 0.791 | -0.172 (-0.485, 0.141) | 0.281 |
| Active commuting to school (Yes) | -0.013 (-0.183, 0.157) | 0.879 | -0.029 (-0.279, 0.221) |  |
| **At age 8 years** | n=235 |  | n=216 |  |
| Organized physical activity (Yes) | **0.222 (0.040, 0.404)** | **0.005** | **0.270 (-0.018, 0.523)** | **0.036** |
| Levels of outdoor active play time |  | **0.021** |  | 0.239 |
| Low | **ref** |  | ref |  |
| Medium | **0.270 (0.067, 0.473)** | **0.009** | 0.240 (-0.045, 0.525) | 0.098 |
| High | **0.231 (0.023, 0.440)** | **0.030** | 0.173 (-0.123, 0.470) | 0.251 |
| Levels of indoor active play time |  | 0.166 |  | 0.074 |
| Low | ref |  | ref |  |
| Medium | -0.032 (-0.224, 0.161) | 0.746 | 0.065 (-0.210, 0.341) | 0.640 |
| High | -0.197 (-0.413, 0.020) | 0.075 | -0.271 (-0.579, 0.037) | 0.084 |
| Active commuting to school (Yes) | 0.050 (-0.112, 0.213) | 0.541 | -0.137 (-0.366, 0.093) | 0.242 |
| MB, movement behaviours; CI, confidence interval  Model 2: adjusted for child sex, ethnicity, BMI at age 5 or 8 years and maternal age and education + accelerometer-measured inactivity and sleep. Organized physical activity, indoor and outdoor active play and active commuting were mutually adjusted.  Significant associations are shown in bold | | | | |

Supplementary Table 3: Prospective associations of parent/caregiver reported domain-specific sedentary behaviour at age 5.5 and 8 years with executive function at age 8.5 years and academic achievement scores at age 9 years in the GUSTO cohort study (Sensitivity analysis based on complete case)

|  | **Executive function at age 8.5 years** | | **Academic achievement at age 9 years** | |
| --- | --- | --- | --- | --- |
|  | **Model 2** | | **Model 2** | |
|  | Mean difference (95% CI) | p-value | Mean difference (95% CI) | p-value |
| **At age 5.5 years** | n=235 |  | 216 |  |
| Total SVT, h/day | 0.027 (-0.030, 0.084) | 0.358 | -0.057 (-0.140, 0.025) | 0.172 |
| Television time, h/day | 0.059 (-0.038, 0.156) | 0.234 | -0.091 (-0.231, 0.048) | 0.197 |
| Handheld devices time, h/day | 0.010 (-0.105, 0.124) | 0.866 | -0.054 (-0.210, 0.102) | 0.495 |
| Playing board/card games (yes) | -0.059 (-0.235, 0.117) | 0.508 | 0.075 (-0.174, 0.323) | 0.553 |
| Sitting and reading books, (h/day) | 0.103 (-0.071, 0.278) | 0.244 | 0.068 (-0.225, 0.361) | 0.648 |
| **At age 8 years** |  |  |  |  |
| Total SVT, h/day | -0.021 (-0.059, 0.018) | 0.290 | **-0.073 (-0.127, -0.019)** | **0.008** |
| Television time, h/day | -0.007 (-0.072, 0.057) | 0.819 | -0.049 (-0.140, 0.042) | 0.293 |
| Handheld devices time, h/day | -0.016 (-0.086, 0.054) | 0.648 | **-0.129 (-0.228, -0.029)** | **0.012** |
| Playing board/card games (yes) | 0.023 (-0.155, 0.201) |  | -0.073- (0.317, 0.171) | 0.555 |
| Sitting and reading books, h/d | **0.220 (0.070, 0.370)** | **0.004** | **0.325 (0.099, 0.551)** | **0.005** |
| MB, movement behaviours; CI, confidence interval  Model 2: adjusted for child sex, ethnicity, BMI at age 5 or 8 years and maternal age and education + accelerometer-measured moderate-to-vigorous physical activity and sleep. Total screen viewing time or television and handheld devices time, playing board/card games and reading books were mutually adjusted  Significant associations are shown in bold | | | | |

Supplementary Figure 3: Estimated changes in the executive function scores associated with hypothetical pairwise reallocation of time from one behaviour to another behaviour at age 5.5 years and 8 years in the GUSTO study


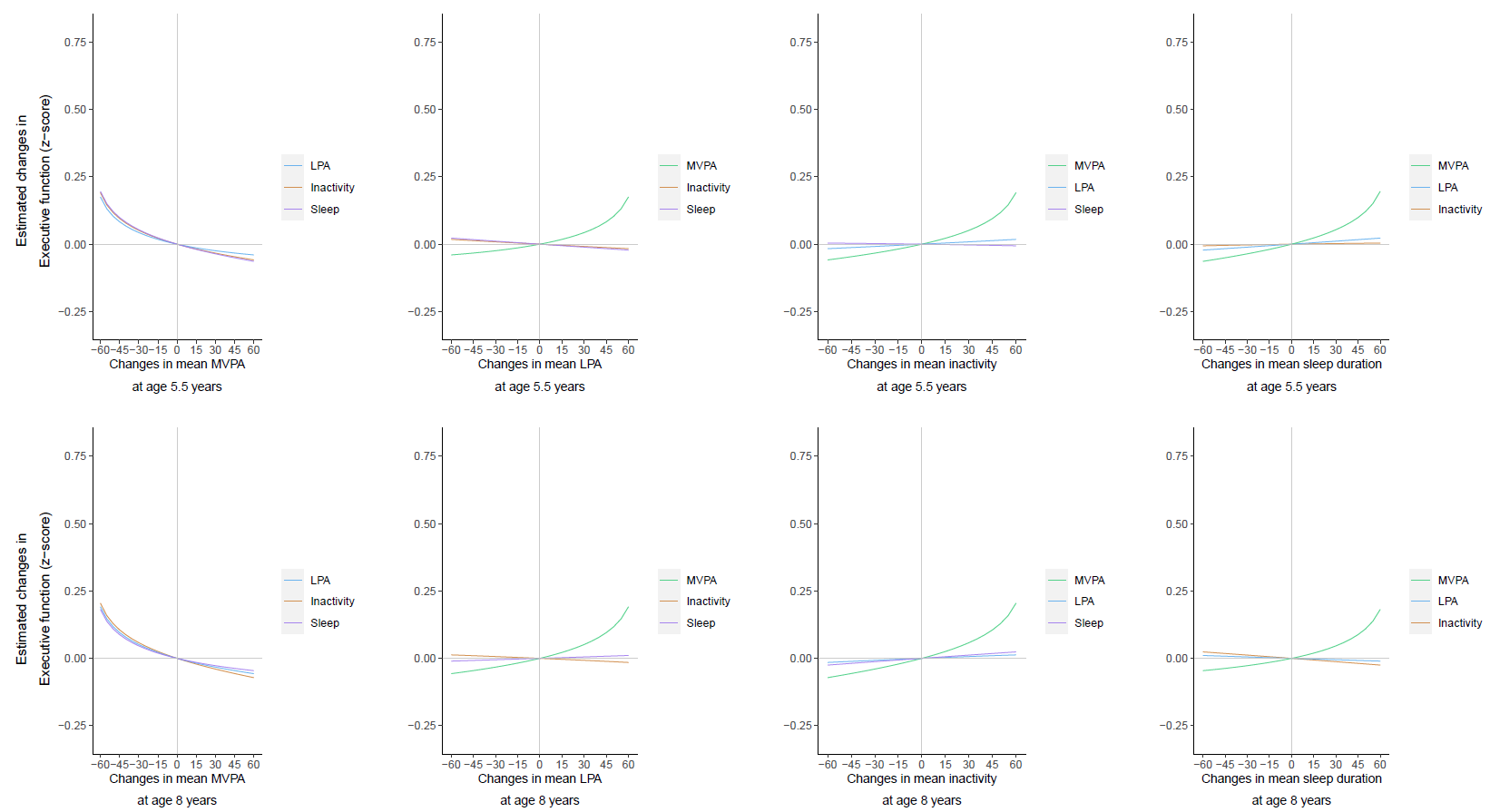


MVPA, moderate-to-vigorous physical activity; Light physical activity (LPA); MB, movement behaviour. Results were based on adjusted for sex, ethnicity, BMI at age 5 or 8 years and maternal age and education. *Corresponding changes in the academic achievement was statistically significant.

Supplementary Figure 4: Estimated changes in the academic achievement scores associated with hypothetical pairwise reallocation of time from one behaviour to another behaviour at age 5.5 years and 8 years in the GUSTO study
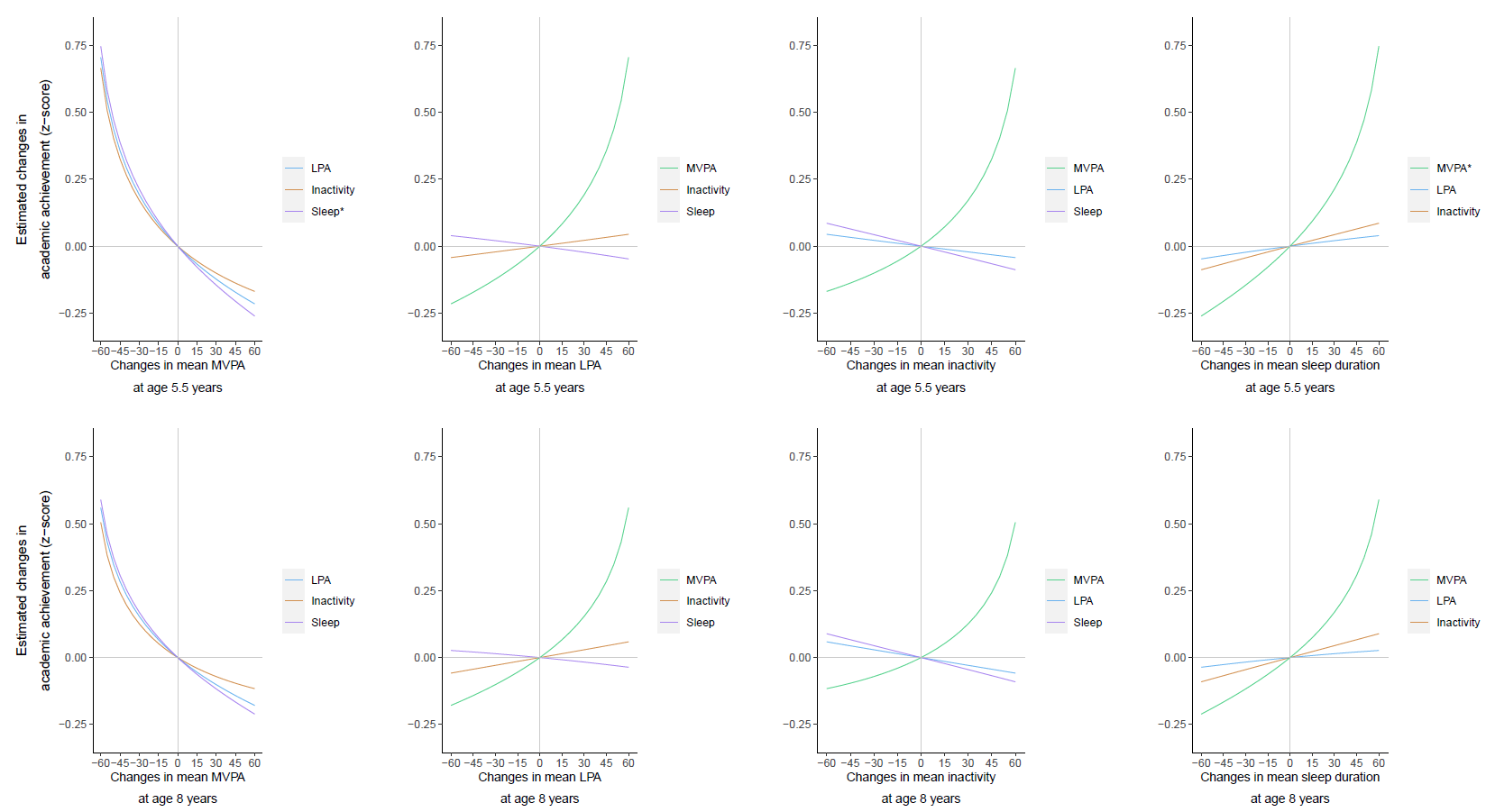


MVPA, moderate-to-vigorous physical activity; Light physical activity (LPA); MB, movement behaviour. Results were based on adjusted for sex, ethnicity, BMI at age 5 or 8 years and maternal age and education. *Corresponding changes in the academic achievement was statistically significant.
